# Health information sources and behaviour changes in a community population: a longitudinal study

**DOI:** 10.1265/ehpm.26-00060

**Published:** 2026-07-14

**Authors:** Yoshitomo Fujita, Sachie Mori, Takehiro Michikawa, Haruhiko Imamura, Minami Sugimoto, Keiko Asakura, Yuji Nishiwaki

**Affiliations:** 1 Department of Environmental and Occupational Health, Toho University Graduate School of Medicine, 5-21-16 Omori-Nishi, Ota, Tokyo 143-8540, Japan; 2 Department of Community Well-Being, School of Medicine, Toho University, 5-21-16 Omori-Nishi, Ota, Tokyo 143-8540, Japan; 3 Department of Environmental and Occupational Health, School of Medicine, Toho University, 5-21-16 Omori-Nishi, Ota, Tokyo 143-8540, Japan; 4 Graduate School of Health and Nutrition Sciences, The University of Nagano, 8-49-7 Miwa, Nagano, Nagano 380-8525, Japan; 5 Department of Preventive Medicine, School of Medicine, Toho University, 5-21-16 Omori-Nishi, Ota, Tokyo 143-8540, Japan

**Keywords:** Health information sources, Behaviour change, Walking time, Balanced meal consumption, Smoking cessation

## Abstract

**Background:**

Accessible and trustworthy health information sources support informed decisions. Longitudinal evidence on how health information sources influence behavioural changes in community-dwelling populations remains limited. Thus, we examined associations between health information sources and behaviour changes in walking time, balanced meal consumption, and smoking cessation.

**Methods:**

Baseline (2021) and follow-up (2023) survey questionnaires were distributed to residents randomly selected from the 18 residential areas. Health information sources were classified into five categories: print media, television (TV) and radio, the Internet, family and friends, and professionals. Desirable health behaviours were defined as walking for ≥60 min/day; consuming ≥2 meals per day comprising staple grains, main dishes, and side items (SMS); and smoking cessation. Meeting these criteria was classified as good; not meeting them as poor. Improvement was defined as poor at baseline and good at follow-up. Maintenance was defined as good at both surveys. Improvement analyses included participants with poor baseline conditions. Maintenance analyses included those with good baseline conditions. Associations were analysed using multilevel modified Poisson regression models, accounting for clustering within residential areas and adjusting for covariates. Risk ratios (RRs) and 95% confidence intervals (95% CIs) were calculated.

**Results:**

Of the 12 119 baseline participants, 6620 (54.6%) completed the follow-up. The most common health information sources were TV and radio (68.5%), the Internet (56.8%), and print media (48.9%). Use of family and friends as information sources was associated with improvement in walking time (RR: 1.19; 95% CI: 1.02–1.39), as was use of professionals (RR: 1.15; 95% CI: 1.03–1.28). Professionals showed a marginal association with sustained walking time (RR: 1.07; 95% CI: 0.99–1.15). TV and radio was associated with improvement in SMS meal consumption (RR: 1.13; 95% CI: 1.02–1.26), while print media was associated with maintenance of SMS meal consumption (RR: 1.05; 95% CI: 1.01–1.08). No sources were related to smoking cessation.

**Conclusions:**

Health information sources showed distinct associations with improved and maintained health behaviours. Interpersonal sources were relevant for improving walking time, while mass media were linked to dietary improvement and maintenance. These findings suggest tailoring health communication strategies to specific health behaviours.

**Supplementary information:**

The online version contains supplementary material available at https://doi.org/10.1265/ehpm.26-00060.

## Graphical Abstract

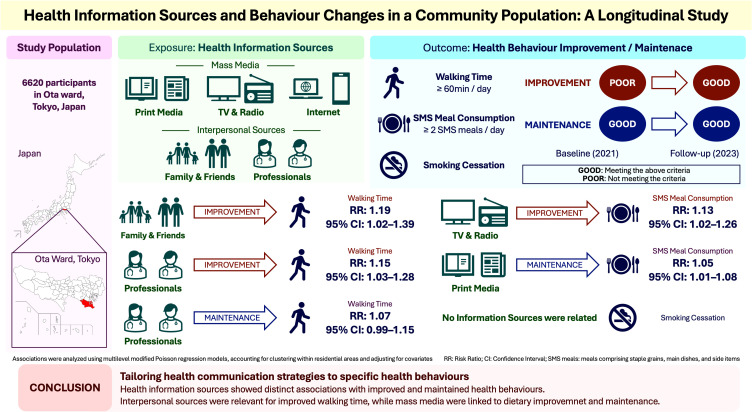

## Background

Accessible and trustworthy health information sources enable individuals to make informed health decisions. Health information users encounter challenges when seeking accurate and relevant information across multiple sources [1]. These sources include mass media, such as newspapers and television (TV); online platforms, including the Internet and social media; and interpersonal channels, such as family, friends, and colleagues. Hospitals and routine health checkups serve as principal providers of health information in healthcare settings. Clearly designated health information sources remain essential, as individuals select different sources based on the specific health behaviour under consideration [2, 3].

These behaviours have been widely examined as key determinants of health: walking time, balanced meal consumption, and smoking cessation [4, 5]. The Japanese Ministry of Health, Labour, and Welfare developed the Physical Activity Guide for Health Promotion 2023. The guide recommends that adults engage in ≥60 min/day of physical activity, including walking or more vigorous exercise [6]. Balanced meal consumption represents a core objective of the third term of Health Japan 21, a national health promotion policy [4, 7]. The policy promotes intake of staple grains, main dishes, and side items, hereafter termed SMS meals, at least twice daily. Adults in Japan who consume SMS meals at least twice per day are more likely to meet the Dietary Reference Intakes compared with those who consume these meals once or less [8, 9]. Smoking causes adverse health outcomes, including cancer, cardiovascular and respiratory diseases, and impaired reproductive outcomes [10]. Additionally, passive smoking contributes to low birth weight, sudden infant death syndrome, and type 2 diabetes mellitus [10]. Therefore, smoking cessation is an essential health behaviour to address.

Previous studies have examined associations between health information sources and health behaviours, including physical activity, diet, and smoking. Redmond et al. have found that use of print media, healthcare workers, and community organisations is linked to diet, physical activity, and smoking cessation [2]. Takaizumi et al. have noted associations among health information sources, dietary behaviour, and physical activity [11]. However, most existing studies have used cross-sectional designs, and few have examined the longitudinal associations between health information sources and health behaviours in community-dwelling populations [12]. Health information sources have changed rapidly over time. Therefore, related evidence must be regularly updated to reflect current trends. Because behavioural change is complex and typically challenging to achieve, providing tailored information is essential.

Personalised advice from family or friends, along with guidance from healthcare professionals based on individual conditions, may promote the improvement and maintenance of health behaviours. Therefore, we hypothesised that the associations between interpersonal and interactive information sources and health behaviours would be stronger than the associations between unidirectional channels, such as television and newspapers, and the same health behaviours. In this study, we examined associations between health information sources and health behaviours, including walking time, SMS meal consumption, and smoking cessation.

## Methods

### Study population and survey questionnaires

This study was conducted in Ota Ward, Tokyo, Japan, as a joint project between the local government of Ota Ward and Toho University [13]. Ota Ward is one of 23 special wards in the Tokyo Metropolitan area, with a population of approximately 740 000. In September 2021, 14-page baseline survey questionnaires were mailed to 36 000 residents aged 20–89 years, with 2 000 individuals randomly selected from each of the 18 residential areas in Ota Ward. Responses were collected via postal mail or online. Each survey questionnaire was assigned an anonymised identifier. Personally identifiable data, such as home addresses or birthdays, were not collected. Both participant enrolment and identifier assignment were conducted in Ota Ward.

**Fig. 1:**
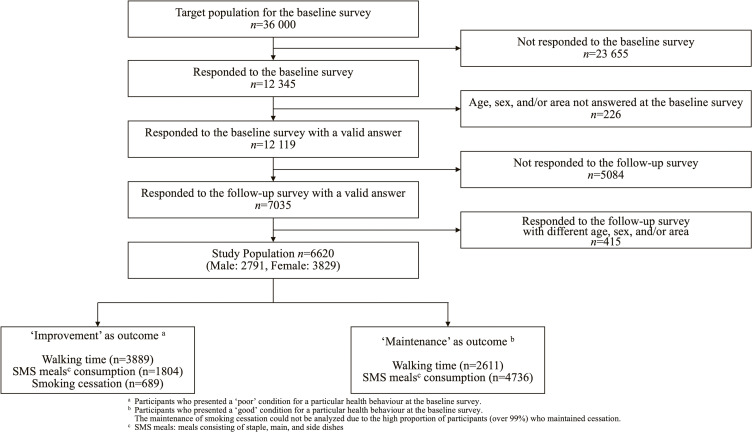
Study Population Flowchart

In total, 12 345 participants responded to the baseline survey questionnaires between October and December 2021. Of these, 12 119 with available data on age, sex, and residential area were included as valid respondents. In September 2023, follow-up survey questionnaires were sent to 36 000 residents, including the 12 119 valid baseline respondents, to ensure comparable sample sizes between the 2021 and 2023 surveys. The final study population comprised 6620 participants after excluding 5084 who did not provide valid follow-up responses and 415 who reported a different age, sex, or area from that of the baseline survey questionnaire. Thus, the follow-up rate was 54.6%, with 6620 of the 12 119 participants completing the survey questionnaires (Fig. 1).

In this study, we administered survey questionnaires to assess physical activity, diet, rest and sleep, smoking status, alcohol consumption, overall health, and health information sources. The findings inform health policy in Ota Ward. Exposure was defined as the health information source reported in the baseline survey questionnaire. Outcomes included improvement and maintenance of health behaviours, including walking time, SMS meal consumption, and smoking cessation.

### Exposure measurement

Participants were asked whether they obtained health information from any sources. Response options included twelve sources along with ‘other’, and ‘none’. The twelve information sources included: (1) books; (2) magazines; (3) newspapers; (4) brochures and flyers; (5) TV; (6) radio; (7) the Internet and smartphone; (8) family; (9) friends and coworkers; (10) physicians and healthcare workers; (11) professionals, such as public health nurses, dietitians, and dental hygienists; and (12) health classes organized by the ward. Responses marked as ‘other’ were reviewed and reclassified into existing categories when appropriate, based on the content of the free-text responses. Participants could select multiple sources. The twelve information sources were classified into five categories according to a previous study [2]: (1) print media, (2) TV and radio, (3) internet, (4) family and friends, and (5) professionals (Table 1). Participants were considered users of a category if they reported using any source within it.

**Table 1:** Five Categories of Health Information Sources

**Category**	**Health Information Sources**
Print Media	• Books • Magazines • Newspapers • Brochures and Flyers
TV and Radio	• TV • Radio
Internet	• Internet and Smartphone
Family and Friends	• Family • Friends and Coworkers
Professionals	• Physicians and Healthcare Workers • Professionals (Public Health Nurses, Dietitians, and Dental Hygienists) • Health Classes Organized by the Ward

TV: television

### Assessment of outcomes

Study outcomes were defined as desirable health behaviours, including walking time, SMS meal consumption, and smoking cessation. For each behaviour, participants were classified as having either a good or poor condition at baseline and follow-up (Supplementary Table 1). Improvement was defined as a transition from a poor condition at baseline to a good condition at follow-up, while maintenance was defined as a good condition at both survey questionnaires.

Walking time was evaluated by asking participants how long they walked on average per day. Response options included: ≥90 min, 60–89 min, 30–59 min, and <30 min. A good condition was defined as walking ≥60 min/day, and a poor condition as walking as <60 min/day, according to the Physical Activity and Exercise Guide by the Ministry of Health, Labour, and Welfare [6].

Balanced meal consumption was assessed by asking participants how many meals per day included staple grains, main dishes, and side items. The response options were three meals, two meals, one meal, or none. A good condition was defined as consuming ≥2 meals per day, and a poor condition as consuming ≤1 meal per day, based on the dietary goals of Health Japan 21, by the Ministry of Health, Labour, and Welfare [7, 8].

Smoking status was assessed by asking participants about their smoking habits. Response options were categorized as ‘has not smoked’, ‘has smoked yet quit’, ‘smokes <one pack per day’, or ‘smokes ≥one pack per day’. A good condition was defined as current non-smoking (participants who had never smoked or had quit), and a poor condition as current smoking.

### Baseline covariates

Baseline covariates included self-reported age; sex; height; weight; alcohol consumption; smoking, living, and marital status; education level; and history of major illness. Smoking status was excluded as a covariate in analyses of smoking cessation. Age was treated as a continuous variable. Body mass index (BMI) was calculated as weight divided by height squared (kg/m^2^) and categorized as: <18.5 kg/m^2^, 18.5–24.9 kg/m^2^, and ≥25.0 kg/m^2^ [14]. A history of major illnesses was defined as a reported history of diabetes mellitus, cerebral stroke, myocardial infarction, angina pectoris, cancer, or related free-text responses. Other covariates were dichotomized as follows: sex (male or female), alcohol consumption (yes or no), smoking status (yes or no), marital status (married or other), education level (university and higher or high school and lower), and living status (living alone or living with others).

### Statistical analysis

Baseline participant characteristics were summarised overall and by sex. Use of health information sources at baseline was described overall and stratified by sex and age groups. Age groups were defined as 20–44, 45–64, and 65–89 years old. Participants aged ≥65 years were classified as older adults, whereas those aged 20–64 years were divided into young adults (20–44 years) and middle-aged (45–64 years) groups for simplicity and interpretability.

Analyses of improvement were restricted to participants in a poor condition at baseline, including walking time (n = 3889), SMS meal consumption (n = 1804), and smoking cessation (n = 689). Analyses of maintenance were restricted to participants in a good condition at baseline, including walking time (n = 2611) and SMS meal consumption (n = 4736).

Associations between the five information categories and improvements in health behaviours were analysed using four models: (1) crude, (2) age- and sex-adjusted, (3) adjusted for all covariates (Model 1), and (4) Model 1 covariates and additional health information sources for mutual adjustment (Model 2). The same modelling strategy was applied to analyse associations with maintenance of health behaviours. Smoking cessation was excluded from maintenance analyses, because >99% of participants remained non-smokers at follow-up.

Using Model 2, stratified analyses by age and sex were conducted to explore differences in associations within strata. Effect modification was assessed by including interaction terms. Due to an unexpected inverse association between use of family and friends as a health information source and maintenance of walking time, additional analyses stratified by living and marital status were performed. Furthermore, age-stratified analyses were conducted for participants aged ≥75 years old.

Multi-level modified Poisson regression models were used to estimate risk ratios (RRs) and 95% confidence intervals (95% CIs), accounting for clustering within the 18 residential areas. The modified Poisson regression approach, which applies robust error variance to Poisson regression, rectified variance overestimation in binary outcomes and allowed direct estimation of RRs [15].

All analyses were performed using STATA version 16.1 (StataCorp, College Station, TX, USA). Statistical significance was set at p < 0.05.

## Results

### Baseline participant characteristics

Of the 6620 participants, 2791 were male and 3829 were female. The mean age (standard deviation) of the study participants at baseline was 59.4 (16.5) years old. Table 2 summarises the baseline participant characteristics. The older age group (65–89 years old) comprised the largest proportion of participants (n = 2828, 42.7%), followed by the middle-aged group (45–64 years old; 36.8%) and young adult group (20–44 years old; 20.5%). Regarding BMI, 3.0% and 14.2% of male and female participants, respectively, were underweight. By contrast, 28.3% and 14.9% of male and female participants, respectively, were obese. Overall, 20.1% of participants reported a history of major illness (males: 26.2%; females: 15.6%). Among participants, 56.3% consumed alcohol (males: 69.4%; females: 46.8%), and 10.4% (males: 16.4%; females: 6.1%) were current smokers. Two-thirds of participants (males: 71.8%; females: 62.8%) were married. A total of 42.6% (males: 56.9%; females: 32.2%) had current university or higher education, and 17.5% (males: 16.7%; females: 18.0%) lived alone.

**Table 2:** Baseline Participant Characteristics

		**Total** **(n = 6620)** **n (%)**	**Male** **Participants** **(n = 2791)** **n (%)**	**Female** **Participants** **(n = 3829)** **n (%)**
Age (years old)	20–44	1354 (20.5)	501 (18.0)	853 (22.3)
	45–64	2438 (36.8)	1024 (36.7)	1414 (36.9)
	65–89	2828 (42.7)	1266 (45.4)	1562 (40.8)

Body Mass Index (kg/m^2^)	<18.5	629 (9.5)	85 (3.0)	544 (14.2)
	18.5–24.9	4536 (68.5)	1886 (67.6)	2650 (69.2)
	≥25.0	1360 (20.5)	790 (28.3)	570 (14.9)
	Missing	95 (1.4)	30 (1.1)	65 (1.7)

History of Major Illness	Yes	1329 (20.1)	731 (26.2)	598 (15.6)
	No	5291 (79.9)	2060 (73.8)	3231 (84.4)

Alcohol Consumption	Yes	3729 (56.3)	1937 (69.4)	1792 (46.8)
	No	2864 (43.3)	840 (30.1)	2024 (52.9)
	Missing	27 (0.4)	14 (0.5)	13 (0.3)

Smoking Status	Yes	691 (10.4)	459 (16.4)	232 (6.1)
	No	5897 (89.1)	2318 (83.1)	3579 (93.5)
	Missing	32 (0.5)	14 (0.5)	18 (0.5)

Marital Status	Married	4411 (66.6)	2005 (71.8)	2406 (62.8)
	Others	2154 (32.5)	769 (27.6)	1385 (36.2)
	Missing	55 (0.8)	17 (0.6)	38 (1.0)

Education	University or higher	2823 (42.6)	1589 (56.9)	1234 (32.2)
	High school or lower	3754 (56.7)	1184 (42.4)	2570 (67.1)
	Missing	43 (0.6)	18 (0.6)	25 (0.7)

Living Status	Living Alone	1158 (17.5)	467 (16.7)	691 (18.0)
	Others	5423 (81.9)	2308 (82.7)	3115 (81.4)
	Missing	39 (0.6)	16 (0.6)	23 (0.6)

### Distribution of health information sources at baseline

Table 3 presents the distribution of health information sources reported by participants at baseline. TV and radio were the most frequently used sources (68.5% of participants), followed by the Internet (56.8%) and print media (48.9%). Patterns were generally consistent across sexes. The Internet was the most commonly used health information source among the young adult and middle-aged groups, while older adults relied primarily on TV and radio. Professionals were the least utilized health information source across most age groups and sexes, apart from older adults.

**Table 3:** Use of Health Information Sources at Baseline

	**Total** **(n = 6620)** **n (%)^a^**	**Male** **Participants** **(n = 2791)** **n (%)^a^**	**Female** **Participants** **(n = 3829)** **n (%)^a^**	**20–44 years old** **(n = 1354)** **n (%)^a^**	**45–64 years old** **(n = 2438)** **n (%)^a^**	**65–89 years old** **(n = 2828)** **n (%)^a^**
1. Print Media	3234 (48.9)	1293 (46.3)	1941 (50.7)	412 (30.4)	1043 (42.8)	1779 (62.9)
2. TV and Radio	4532 (68.5)	1735 (62.2)	2797 (73.1)	696 (51.4)	1652 (67.8)	2184 (77.2)
3. Internet	3758 (56.8)	1521 (54.5)	2237 (58.4)	1144 (84.5)	1836 (75.3)	778 (27.5)
4. Family and Friends	2282 (34.5)	791 (28.3)	1491 (38.9)	493 (36.4)	799 (32.8)	990 (35.0)
5. Professionals	1662 (25.1)	752 (26.9)	910 (23.8)	213 (15.7)	530 (21.7)	919 (32.5)

^a^Number and percentage of participants who have responded ‘yes’ to the use of health information sources from each category. Totals exceed 100%, due to multiple responses.

TV: television.

### Associations between health information sources and behaviour changes

Table 4 presents the associations between health information sources and improvements in health behaviours. Participants who used family and friends or professionals as health information sources were more likely to show improvements in walking time across all four models. In Model 2, which adjusted for all covariates and simultaneously included all five health information categories, the adjusted RR for family and friends was 1.19 (95% CI: 1.02–1.39) and for professionals was 1.15 (95% CI: 1.03–1.28). Stratified analyses by sex and age (Supplementary Fig. 1d) showed that the association between family and friends and improvement in walking time was consistent across groups, with no notable effect modification. Conversely, the association regarding professionals was more pronounced among male participants (RR: 1.25; 95% CI: 1.06–1.47) and the young adult group (RR: 1.53; 95% CI: 1.15–2.03; Supplementary Fig. 1e). However, effect modification by sex and age was not meaningful.

**Table 4:** Association Between Health Information Sources and Improvement in Health Behaviours

		**Use of** **Information**	**Improved** **/n**	**(%)**	**Crude**		**Age and sex-adjusted**		**Model 1^a^**		**Model 2^b^**	
			
					**RR**	**95%CI**	**RR**	**95%CI**	**RR**	**95%CI**	**RR**	**95%CI**
Walking Time	Print Media	yes	434/1766	(24.6)	1.17*	1.03–1.33	1.15*	1.01–1.31	1.15^#^	0.99–1.33	1.10	0.95–1.27
		no	447/2123	(21.1)	1.00		1.00		1.00		1.00	
	TV and Radio	yes	615/2577	(23.9)	1.18^#^	0.99–1.39	1.14	0.97–1.33	1.14	0.97–1.35	1.08	0.92–1.27
		no	266/1312	(20.3)	1.00		1.00		1.00		1.00	
	Internet	yes	519/2260	(23.0)	1.03	0.89–1.20	1.06	0.92–1.23	1.05	0.89–1.24	1.02	0.86–1.20
		no	362/1629	(22.2)	1.00		1.00		1.00		1.00	
	Family and Friends	yes	336/1253	(26.8)	1.30*	1.13–1.49	1.26*	1.09–1.46	1.23*	1.05–1.45	1.19*	1.02–1.39
		no	545/2636	(20.7)	1.00		1.00		1.00		1.00	
	Professionals	yes	233/926	(25.2)	1.15*	1.04–1.28	1.16*	1.04–1.29	1.18*	1.05–1.33	1.15*	1.03–1.28
		no	648/2963	(21.9)	1.00		1.00		1.00		1.00	

SMS Meal Consumption	Print Media	yes	353/720	(49.0)	1.12*	1.00–1.26	1.11^#^	0.99–1.24	1.12^#^	0.99–1.25	1.08	0.96–1.21
		no	473/1084	(43.6)	1.00		1.00		1.00		1.00	
	TV and Radio	yes	563/1156	(48.7)	1.20*	1.08–1.34	1.17*	1.05–1.31	1.16*	1.05–1.29	1.13*	1.02–1.26
		no	263/648	(40.6)	1.00		1.00		1.00		1.00	
	Internet	yes	516/1101	(46.9)	1.06	0.95–1.18	1.11	0.97–1.28	1.05	0.90–1.21	1.03	0.89–1.19
		no	310/703	(44.1)	1.00		1.00		1.00		1.00	
	Family and Friends	yes	273/574	(47.6)	1.06	0.93–1.20	1.04	0.92–1.18	1.02	0.89–1.17	0.99	0.86–1.14
		no	553/1230	(45.0)	1.00		1.00		1.00		1.00	
	Professionals	yes	181/372	(48.7)	1.08	0.98–1.19	1.08	0.97–1.19	1.05	0.95–1.17	1.04	0.94–1.15
		no	645/1432	(45.0)	1.00		1.00		1.00		1.00	

Smoking Cessation	Print Media	yes	31/232	(13.4)	1.39*	1.05–1.83	1.15	0.83–1.60	1.06	0.78–1.42	1.03	0.72–1.47
		no	44/457	(9.6)	1.00		1.00		1.00		1.00	
	TV and Radio	yes	50/417	(12.0)	1.30	0.75–2.26	1.17	0.67–2.04	1.10	0.61–1.97	1.10	0.59–2.05
		no	25/272	(9.2)	1.00		1.00		1.00		1.00	
	Internet	yes	39/379	(10.3)	0.89	0.54–1.46	1.27	0.79–2.04	1.09	0.65–1.83	1.12	0.67–1.87
		no	36/310	(11.6)	1.00		1.00		1.00		1.00	
	Family and Friends	yes	18/200	(9.0)	0.77	0.50–1.18	0.80	0.51–1.25	0.82	0.52–1.30	0.80	0.52–1.24
		no	57/489	(11.7)	1.00		1.00		1.00		1.00	
	Professionals	yes	21/169	(12.4)	1.20	0.79–1.81	1.04	0.67–1.61	0.93	0.57–1.52	0.95	0.59–1.52
		no	54/520	(10.4)	1.00		1.00		1.00		1.00	

RR: risk ratio; CI: confidence interval; SMS meals: staple grains, main dishes, and side items.

*p < 0.05

^#^p < 0.1

^a^Adjusted for age; sex; body mass index; history of major illness; alcohol consumption; education level; marital, living, and smoking status. Excludes smoking status when smoking cessation serves as the outcome.

^b^Adjusted for all the covariates in Model 1 and additional health information sources.

A consistent association was observed between TV and radio use and improved SMS meal consumption, suggesting that participants who relied on these health information sources were more likely to better their eating habits. The adjusted RR (Model 2) was 1.13 (95% CI: 1.02–1.26). Stratified analyses showed no distinct effect modifications by sex or age group. Nonetheless, the association appeared stronger among female participants (RR: 1.18; 95% CI: 1.02–1.36) and the middle-aged group (RR: 1.24; 95% CI: 1.01–1.51; Supplementary Fig. 2b).

No associations were found between any health information source and smoking cessation in either overall or stratified analyses (Supplementary Fig. 3).

### Health information sources and behaviour maintenance

Table 5 shows the association between health information sources and maintenance of health behaviours. Regarding walking time, the use of professionals was marginally associated with a higher likelihood of maintenance in Model 2 (RR: 1.07; 95% CI: 0.99–1.15). This association was mainly driven by the young adult group (RR: 1.26; 95% CI: 1.08–1.48), with a statistically significant age interaction (p = 0.034; Supplementary Fig. 4e). Unexpectedly, participants who used family and friends as a health information source were less likely to maintain walking time (RR: 0.95; 95% CI: 0.91–1.00). This inverse association was most evident among participants who were not living alone (RR: 0.93; 95% CI: 0.87–0.99) or who were married (RR: 0.91; 95% CI: 0.85–0.98; Supplementary Fig. 5). An inverse association was further noted in the group aged ≥75 years old (RR: 0.90; 95% CI: 0.79–1.03). However, this was not statistically significant (Supplementary Fig. 5).

**Table 5:** Association Between Health Information Sources and Maintenance of Health Behaviours

		**Use of** **Information**	**Maintained** **/n**	**(%)**	**Crude**		**Age and sex-adjusted**		**Model 1^a^**		**Model 2^b^**	
			
					**RR**	**95%CI**	**RR**	**95%CI**	**RR**	**95%CI**	**RR**	**95%CI**
Walking Time	Print Media	yes	949/1428	(66.5)	1.03	0.97–1.09	1.02	0.96–1.09	1.04	0.98–1.11	1.03	0.97–1.10
		no	764/1183	(64.6)	1.00		1.00		1.00		1.00	
	TV and Radio	yes	1257/1896	(66.3)	1.04	0.97–1.11	1.03	0.97–1.10	1.04	0.97–1.11	1.03	0.96–1.11
		no	456/715	(63.8)	1.00		1.00		1.00		1.00	
	Internet	yes	972/1471	(66.1)	1.02	0.95–1.08	1.04	0.98–1.10	1.04	0.98–1.11	1.04	0.98–1.11
		no	741/1140	(65.0)	1.00		1.00		1.00		1.00	
	Family and Friends	yes	645/1004	(64.2)	0.97	0.93–1.01	0.96^#^	0.93–1.01	0.97	0.92–1.01	0.95^#^	0.91–1.00
		no	1068/1607	(64.8)	1.00		1.00		1.00		1.00	
	Professionals	yes	482/712	(59.4)	1.04	0.97–1.13	1.04	0.96–1.13	1.07^#^	0.99–1.15	1.07^#^	0.99–1.15
		no	1231/1899	(52.6)	1.00		1.00		1.00		1.00	

SMS Meal Consumption	Print Media	yes	2137/2485	(86.0)	1.09*	1.06–1.12	1.07*	1.04–1.10	1.05*	1.02–1.08	1.05*	1.01–1.08
		no	1778/2251	(79.0)	1.00		1.00		1.00		1.00	
	TV and Radio	yes	2798/3335	(83.9)	1.05*	1.02–1.09	1.03	0.99–1.06	1.02	0.99–1.06	1.01	0.97–1.05
		no	1117/1401	(79.7)	1.00		1.00		1.00		1.00	
	Internet	yes	2170/2645	(82.0)	0.98	0.96–1.01	1.04*	1.01–1.07	1.03	0.99–1.06	1.02	0.99–1.05
		no	1745/2091	(83.5)	1.00		1.00		1.00		1.00	
	Family and Friends	yes	1433/1692	(84.7)	1.04*	1.02–1.06	1.03*	1.01–1.05	1.02^#^	0.99–1.04	1.01	0.99–1.03
		no	2482/3044	(81.5)	1.00		1.00		1.00		1.00	
	Professionals	yes	1065/1266	(84.1)	1.02^#^	0.99–1.05	1.01	0.98–1.04	1.01	0.98–1.03	1.00	0.98–1.03
		no	2850/3470	(82.1)	1.00		1.00		1.00		1.00	

RR: risk ratio; CI: confidence interval; SMS meals: staple grains, main dishes, and side items.

*p < 0.05

^#^p < 0.1

^a^Adjusted for age; sex; body mass index; history of major illness; alcohol consumption; education level; marital, living, and smoking status.

^b^Adjusted for all covariates in Model 1 and additional health information sources.

The use of print media was consistently associated with better maintenance of SMS meal consumption across all four models, with an adjusted RR of 1.05 (95% CI: 1.01–1.08) in Model 2. Stratified analysis indicated the association was largely attributable to female participants (RR: 1.07; 95% CI: 1.04–1.11; Supplementary Fig. 6a). Maintenance of smoking cessation was not analysed, because >99% of participants had maintained cessation, leaving insufficient variability for meaningful analysis.

## Discussion

In this study, improvement and maintenance of walking time were associated with the interpersonal and interactive health information sources, including family and friends or professionals. By contrast, improvement and maintenance of SMS meal consumption were linked to unidirectional sources, such as TV, radio and print media.

The association between family and friends and improved walking time highlights the value of interpersonal information. Additionally, family and friends may offer encouragement and practical support, which may explain the consistent associations across age and sex groups. The observed improvement in walking time reflects a transition from the precontemplation, contemplation or preparation stage to the action stage in the Transtheoretical Model [16]. Personalised advice and emotional support may have facilitated behaviour change through processes, such as consciousness raising, which increases awareness of benefits, and dramatic relief, which involves emotional reactions to perceived health risks [17]. Observing family and friends walking regularly may have further promoted behaviour change via social learning [18]. The association regarding professionals suggests that individualised instructions from healthcare workers contributed to improvement in walking time, particularly among younger adults, who generally had fewer opportunities to interact with healthcare workers than did older adults. For younger adults, advice received through follow-up or management of medical conditions may have been novel and impactful, prompting behavioural change. Consistent with this interpretation, the association between professionals and maintenance of walking time was primarily observed in the young adult group. Conversely, an inverse association was found between family and friends and maintenance of walking time. Stratified analyses indicated this inverse association occurred among participants aged ≥75 years old, those not living alone, and those who were married. These populations are more likely to experience frailty, and individuals with frailty may rely on family or friends as health information sources. Thus, the observed inverse association may reflect the inclusion of frail participants who obtained information from family and friends. Model 2 was adjusted for a history of major diseases. However, frailty was not directly measured, and adjustments for major diseases may not fully account for its effects.

Associations between health information sources and physical activity have been examined in previous studies. Cross-sectional studies have shown that newspapers, books, magazines, professional advice, and participation in health classes are associated with physical activity for cancer prevention [19]. Another study found that the use of magazines and the Internet was linked to engaging in ≥23 METs of physical activity per week [11]. Conversely, no clear associations were observed between information sources and physical activity in a study using data from the National Health Information Survey in the United States of America [2]. Overall, the evidence remains inconsistent, likely due to differences in study design, definitions and measurements of physical activity, and population characteristics.

The association between TV and radio and improvement in SMS meal consumption suggests the potential effectiveness of unidirectional health information sources. TV is unidirectional yet delivers both audio and visual information, which may enhance message persuasiveness and facilitate processes, such as consciousness raising and dramatic relief as per the Transtheoretical Model. In Japan, TV is the most frequently used source of dietary information, supporting its potential role in dietary behaviour changes [20, 21]. Interaction terms did not show a clear effect. Nonetheless, stratified analyses showed stronger associations among female and middle-aged participants. These participants were more likely to prepare meals and more frequently exposed to dietary information via TV and radio. Print media was associated with maintenance of SMS meal consumption. Print media is a unidirectional channel that allows repeated access to information. This repeated access may facilitate stimulus control, which is the use of environmental cues to recall and sustain behaviour change. Stimulus control is a process in the Transtheoretical Model [17]. Similarly, previous studies have found associations between unidirectional sources, including books, magazines, websites, and balanced diets [11, 19]. However, findings regarding interpersonal sources have been inconsistent in previous studies. Some studies have observed associations between professionals or health classes and balanced dietary behaviours, particularly in cancer prevention [19]. Other studies have found associations with family members based on relatively small samples [11]. Outcome definitions varied across studies. One study identified an association only with information from community organisations, using a strict dietary guideline adherence outcome met by 11% of participants, compared with 72% meeting the baseline criterion in the present study [2].

Interpersonal and interactive channels, such as family and friends and professionals, were associated with improvement and maintenance of walking time yet not with improvement or maintenance of SMS meal consumption. This difference may reflect the nature of the behaviours. Walking typically requires specific and individualised guidance, such as supportive methods, pace, timing, and location, which is more effectively conveyed through interpersonal communication. Conversely, consuming SMS meals is a common dietary concept in Japan and may be sufficiently supported by repeated exposure to general messages through unidirectional sources.

No health information source predicted smoking cessation in our 2-y longitudinal study. Cross-sectional data from Japan show higher cessation or reduction among individuals who obtain health information sources from healthcare settings, shared personal experiences, and physicians than among those who do not [22]. General recommendations may be insufficient. Specialised programmes may be required. During the study period, detection of carcinogen contamination led to the suspension of varenicline and closure of many smoking cessation clinics. Individuals seeking professional guidance may have encountered reduced access to effective cessation support. The survey questionnaire did not assess clinic use, which prevented evaluation of this factor.

This study has several strengths. First, a longitudinal design with survey questionnaires in 2021 and 2023 enabled analyses of improvements and maintenance of health behaviours. Prior research on health information sources and behaviours has been largely cross-sectional, with few longitudinal studies. Second, the sample included community-dwelling adults, a population with limited epidemiological data. Third, the study population was large; 6620 respondents aged 20–89 years old supported stratified analyses by sex and age. Fourth, analyses adjusted for major confounders, including history of major illnesses and living and marital status. Several limitations warrant consideration. First, older adults were overrepresented, which limits generalizability to the broader population. Thus, age-stratified analyses were conducted. Second, participants who completed both surveys may have been more health-conscious. Consistent with this, obesity prevalence in our sample was lower than national estimates from the 2019 Japan National Health and Nutrition Survey: 28.3% of males and 14.9% of females were classified as obese, compared with 33.0% and 22.3%, respectively [21]. The prevalence of smoking in our sample was also lower than that reported in the 2019 Japan National Health and Nutrition Survey: 16.4% of males and 6.1% of females were current smokers in our study, compared with 27.1% and 7.6%, respectively [21]. Thus, the findings may not be generalizable to individuals with lower health awareness. Third, the survey questionnaires did not assess frequency of access to health information sources or level of comprehension. Effective delivery methods could not be identified. Fourth, self-reported data may have introduced misclassification, likely non-differential, which would attenuate associations. In addition, because participants could select multiple health information sources, some overlap among categories may exist (Supplementary Table 2). The phi coefficients indicated generally weak correlations between sources (e.g., phi = 0.31 between print media and TV and radio, Supplementary Table 3). Although we adjusted for all information sources simultaneously, correlation between sources may not be fully eliminated; therefore, the findings should be interpreted with some caution. Fifth, multiple comparisons increased risk of type I error. No adjustment was applied to preserve statistical power for exploratory analyses. Sixth, the observed associations may reflect not only the potential influence of health information sources but also differences in participants’ underlying health consciousness.

Communication strategies may require alignment of channels with targeted health behaviours. Use of health information sources varied by channel and requires attention. Family and friends were used less frequently, at approximately 30% of all age groups. Professionals were used even less frequently, particularly among younger adults, at 15%. Conversely, unidirectional channels, such as TV, radio, and print media were widely used. No association was observed between the Internet and health behaviours, despite expectations of higher utilization. Further research should be conducted to examine effective communication strategies in community settings, including suburban areas.

## Conclusions

Communication through family and friends and through professionals was associated with increased walking time. TV and radio were associated with increased SMS meal consumption. No health information source was associated with smoking cessation. For maintenance of health behaviours, professional sources showed a marginal association with sustained walking time, and print media was associated with continued SMS meal consumption. These findings support tailoring health communication strategies to specific health behaviours.

## Abbreviations

BMI: body mass index
CI: confidence interval
RR: risk ratio
SMS meals: staple grains, main dishes, and side items
TV: television

## Supplementary information

**Additional file 1:** **Supplementary Table 1:** Definition of Good and Poor Conditions. **Supplementary Figure 1:** Association Between Health Information Sources and Improvement in Walking Time. **Supplementary Figure 2:** Association Between Health Information Sources and Improvement in SMS Meal Consumption. **Supplementary Figure 3:** Association Between Health Information Sources and Smoking Cessation. **Supplementary Figure 4:** Association Between Health Information Sources and Maintenance of Walking Time. **Supplementary Figure 5:** Association Between Family and Friends and Maintenance of Walking Time. **Supplementary Figure 6:** Association Between Health Information Sources and Maintenance of SMS Meal Consumption. **Supplementary Table 2:** Overlap between Health Information Sources. **Supplementary Table 3:** Pairwise Associations (Phi Coefficients) between Health Information Source Categories.

## Declarations

### Availability of data and materials

The data analysed in this study are not publicly available. Data cannot be shared due to privacy and ethical restrictions.
