# Supplementary material for: Health information sources and behaviour changes in a community population: a longitudinal study
Source: Environ Health Prev Med. 2026 Jul 14;31:48. doi: 10.1265/ehpm.26-00060 (PMC13413660; doi:10.1265/ehpm.26-00060)
Supplement: Supplementary file 1 — Additional file 1: Supplementary Table 1: Definition of Good and Poor Conditions. Supplementary Figure 1: Association Between Health Information Sources and Improvement in Walking Time. Supplementary Figure 2: Association Between Health Information Sources and Improvement in SMS Meal Consumption. Supplementary Figure 3: Association Between Health Information Sources and Smoking Cessation. Supplementary Figure 4: Association Between Health Information Sources and Maintenance of Walking Time. Supplementary Figure 5: Association Between Family and Friends and Maintenance of Walking Time. Supplementary Figure 6: Association Between Health Information Sources and Maintenance of SMS Meal Consumption. Supplementary Table 2: Overlap between Health Information Sources. Supplementary Table 3: Pairwise Associations (Phi Coefficients) between Health Information Source Categories. [file ehpm-31-048-s001.docx]

**ADDITIONAL FILE 1**

Supplementary Table 1: Definitions of Good and Poor Conditions

|  | Good | Poor |
| --- | --- | --- |
| Walking Time | - 60–89 min - ≥ 90 min | - 30–59 min - < 30 min |
| Balanced Meal Consumption | - Three meals per day - Two meals per day | - One meal per day - None |
| Smoking Cessation | - Has not smoked - Has smoked yet quit | - Smokes < one pack per day - Smokes ≥ one pack per day |

| p= 0.425 |  | |  |
| --- | --- | --- | --- |
| (a) Print Media | (b) TV and Radio | | (c) Internet |
|  | |  | |
| (d) Family and Friends | | (e) Professionals | |

# Supplementary Figure 1: Association Between Health Information Sources and Improvement in Walking Time

#

RR: risk ratio; TV: television.

^a^ Adjusted for age; sex; body mass index; history of major illness; alcohol consumption; education level; smoking, marital, and living status; and additional health information sources (Model 2).

^b^ All p values shown in the figure correspond with interaction terms.

|  |  | |  |
| --- | --- | --- | --- |
| (a) Print Media | (b) TV and Radio | | (c) Internet |
|  | |  | |
| (d) Family and Friends | | (e) Professionals | |

# Supplementary Figure 2: Association Between Health Information Sources and Improvements in SMS Meal Consumption

RR: Risk Ratio; TV: television

^a^ SMS meals: staple grains, main dishes, and side items

^b^ Adjusted for age; sex; body mass index; history of major illness; alcohol consumption; education level; smoking, marital, and living status; and additional health information sources (Model 2).

^c^ All p values shown in the figure correspond to interaction terms.

|  |  | |  |
| --- | --- | --- | --- |
| (a) Print Media | (b) TV and Radio | | (c) Internet |
|  | |  | |
| (d) Family and Friends | | (e) Professionals | |

# Supplementary Figure 3: Association Between Health Information Sources and Smoking Cessation

RR: Risk Ratio; TV: television.

^a^ Adjusted for age; sex; body mass index; history of major illness; alcohol consumption; education level; smoking, marital, and living status; and additional health information sources (Model 2).

^c^ All p values shown in the figure correspond to interaction terms.

|  |  | |  |
| --- | --- | --- | --- |
| (a) Print Media | (b) TV and Radio | | (c) Internet |
|  | |  | |
| (d) Family and Friends | | (e) Professionals | |

# Supplementary Figure 4: Association Between Health Information Sources and Maintenance of Walking Time

#

RR: risk ratio; TV: television.

^a^ Adjusted for age; sex; body mass index; history of major illness; alcohol consumption; education level; smoking, marital, and living status; and additional health information sources (Model 2).

^b^ All p values shown in the figure correspond with interaction terms.

# Supplementary Figure 5: Association Between Family and Friends and Maintenance of Walking Time

RR: risk ratio; TV: television.

^a^ Adjusted for age; sex; body mass index; history of major illness; alcohol consumption; education level; smoking, marital, and living status; and additional health information sources (Model 2).

^b^ All p values shown in the figure correspond with interaction terms.

|  |  | |  |
| --- | --- | --- | --- |
| (a) Print Media | (b) TV and Radio | | (c) Internet |
|  | |  | |
| (d) Family and Friends | | (e) Professionals | |

# Supplementary Figure 6: Association Between Health Information Sources and Maintenance of SMS Meal Consumption

RR: Risk Ratio; TV: television

^a^ SMS meals: staple grains, main dishes, and side items

^b^ Adjusted for age; sex; body mass index; history of major illness; alcohol consumption; education level; smoking, marital, and living status; and additional health information sources (Model 2).

^c^ All p values shown in the figure correspond to interaction terms.

# Supplementary Table 2: Overlap between Health Information Sources

|  |  | n |
| --- | --- | --- |
| **Print Media** | Total | 3234 |
|  | Solo information source | 92 |
|  | In combination with TV and Radio | 2685 |
|  | In combination with Internet | 1695 |
|  | In combination with Family and Friends | 1272 |
|  | In combination with Professionals | 962 |
|  |  |  |
| **TV and Radio** | Total | 4532 |
|  | Solo information source | 323 |
|  | In combination with Print Media | 2685 |
|  | In combination with Internet | 2545 |
|  | In combination with Family and Friends | 1773 |
|  | In combination with Professionals | 1214 |
|  |  |  |
| **Internet** | Total | 3758 |
|  | Solo information source | 496 |
|  | In combination with Print Media | 1695 |
|  | In combination with TV and Radio | 2545 |
|  | In combination with Family and Friends | 1422 |
|  | In combination with Professionals | 869 |
|  |  |  |
| **Family and Friends** | Total | 2282 |
|  | Solo information source | 61 |
|  | In combination with Print Media | 1272 |
|  | In combination with TV and Radio | 1773 |
|  | In combination with Internet | 1422 |
|  | In combination with Professionals | 730 |
|  |  |  |
| **Professionals** | Total | 1662 |
|  | Solo use | 112 |
|  | In combination with Print Media | 962 |
|  | In combination with TV and Radio | 1214 |
|  | In combination with Internet | 869 |
|  | In combination with Family and Friends | 730 |

# Supplementary Table 3: Pairwise Associations (Phi Coefficients) between Health Information Source Categories

|  | **Print Media** | **TV and Radio** | **Internet** | **Family and Friends** | **Professionals** |
| --- | --- | --- | --- | --- | --- |
| **Print Media** | 1.00 |  |  |  |  |
| **TV and Radio** | 0.31 | 1.00 |  |  |  |
| **Internet** | -0.09 | -0.02 | 1.00 |  |  |
| **Family and Friends** | 0.10 | 0.14 | 0.08 | 1.00 |  |
| **Professionals** | 0.10 | 0.06 | -0.05 | 0.12 | 1.00 |
